# The Intricate Interplay between APOBEC3 Proteins and DNA Tumour Viruses

**DOI:** 10.3390/pathogens13030187

**Published:** 2024-02-20

**Authors:** Nika Lovšin, Bhavani Gangupam, Martina Bergant Marušič

**Affiliations:** 1 Faculty of Pharmacy, University of Ljubljana, Aškerčeva 7, 1000 Ljubljana, Slovenia; marija.nika.lovsin@ffa.uni-lj.si; 2 Laboratory for Environmental and Life Sciences, University of Nova Gorica, Vipavska 13, 5000 Nova Gorica, Slovenia; bhavani.gangupam@ung.si

**Keywords:** APOBEC, DNA tumour viruses, viral restriction, tumorigenesis, DNA editing

## Abstract

APOBEC3 proteins are cytidine deaminases that play a crucial role in the innate immune response against viruses, including DNA viruses. Their main mechanism for restricting viral replication is the deamination of cytosine to uracil in viral DNA during replication. This process leads to hypermutation of the viral genome, resulting in loss of viral fitness and, in many cases, inactivation of the virus. APOBEC3 proteins inhibit the replication of a number of DNA tumour viruses, including herpesviruses, papillomaviruses and hepadnaviruses. Different APOBEC3s restrict the replication of different virus families in different ways and this restriction is not limited to one APOBEC3. Infection with DNA viruses often leads to the development and progression of cancer. APOBEC3 mutational signatures have been detected in various cancers, indicating the importance of APOBEC3s in carcinogenesis. Inhibition of DNA viruses by APOBEC3 proteins appears to play a dual role in this process. On the one hand, it is an essential component of the innate immune response to viral infections, and, on the other hand, it contributes to the pathogenesis of persistent viral infections and the progression of cancer. The current review examines the complex interplay between APOBEC3 proteins and DNA viruses and sheds light on the mechanisms of action, viral countermeasures and the impact on carcinogenesis. Deciphering the current issues in the interaction of APOBEC/DNA viruses should enable the development of new targeted cancer therapies.

## 1. Introduction

APOBEC3 (A3) proteins are cytidine deaminases that belong to the family of apolipoprotein B mRNA editing complex polypeptide 1–like (APOBEC) enzymes. Originally discovered as antiviral factors in HIV-1 infection [1], APOBEC3s were later identified as general antiviral factors that block the replication of various viruses and retrotransposons [2,3,4]. Intriguingly, recent broad genomic studies have uncovered APOBECs as an important source of somatic mutations in various cancers, including breast, ovarian, bladder, prostate, pancreatic, kidney, and cervical cancers [4] and thus as a possible mechanism contributing to cell transformation and cancer development.

The *APOBEC3* genes belong to the interferon-stimulated genes (ISGs). During viral infections, the expression of interferons (IFNs) and cytokines/chemokines is controlled by cytosolic nucleic acid sensors that recognize foreign RNA and DNA [5]. When the innate immune system responds to viral infections, particularly at mucosal sites where sentinel cells such as macrophages and dendritic cells (DCs) are crucial, there is a boost in APOBEC3 activity [3]. Although the regulation of *APOBEC3* expression is not yet fully understood, this interplay between innate immune sensors and APOBEC3 provides the host with multi-layered protection against infection by different virus families. Co-evolution between virus and host leads to the development of different strategies by which to limit the APOBEC3 restriction. Active strategies involve viral proteins capable of degrading or relocalising APOBEC3 [6,7]. In contrast, a passive strategy usually involves depletion of APOBEC3-favoured motifs from the viral genome, which is referred to as the evolutionary footprint of APOBEC3 [8].

Most early research on the antiviral activity of APOBEC3 has focused on retroviruses and retroelements, with deamination of retroviral RNA being the primary mechanism of viral inhibition [9]. However, APOBEC3 proteins are also strongly involved in the inhibition of DNA viruses. The genome of DNA viruses becomes a compelling substrate for APOBEC3 deamination mostly during replication and transcription, resulting in single-stranded DNA. However, APOBEC3 editing is not restricted to the DNA genome. This intrinsic mechanism of host defence may also be responsible for a long-term host’s DNA hypermutation and cancer development during persistent viral infections.

Infection with oncogenic viruses causes about 15–20% of all cancers in humans, and DNA tumour viruses account for a large proportion of these [10]. The human papillomavirus (HPV), the hepatitis B virus (HBV) and the Epstein–Barr virus (EBV) alone are responsible for 85% of all cancers caused by viral infections [11]. Some of these viruses encode oncogenes that target host tumour suppressor genes [12]; however, at least part of cancer development depends on somatic mutagenesis that targets specific host genes involved in cell proliferation and survival. APOBEC3 proteins are crucial players in the interaction of DNA viruses with their human hosts and may represent a viable target for diagnostic or therapeutic intervention. In this review, we aim to highlight the fine line between the antiviral activity of APOBEC3 proteins and deleterious host DNA editing when this process is deregulated.

## 2. APOBEC Proteins and Their Role in Viral Inhibition and Genome Editing

**Figure 1:**
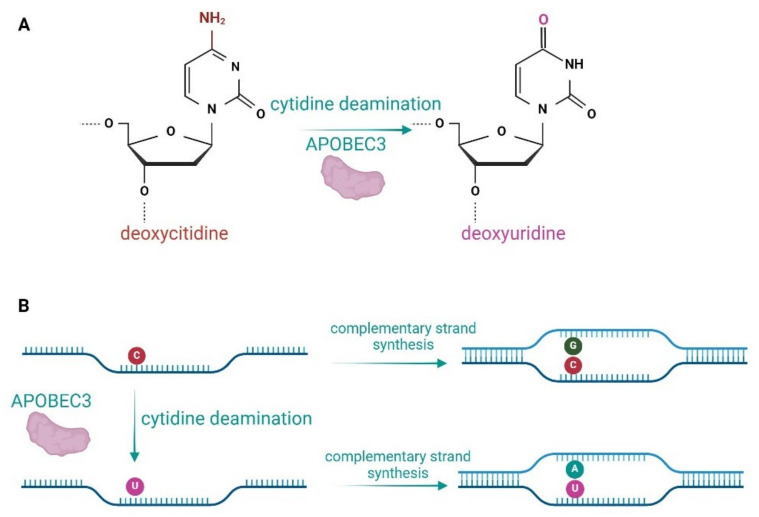
Enzymatic activity of APOBEC3 proteins. (**A**) APOBEC3s deaminate deoxycytidines to deoxyuridines in ssDNA. (**B**) Deamination of deoxycytidines in the lagging DNA strand leads to a G-to-A mutation in complementary strand synthesis.

The APOBEC enzymes are able to edit DNA and RNA [1,13,14]. They deaminate cytosine to uracil (C-to-U) in a single-stranded nucleic acid, which in the case of DNA causes a G-to-A mutation in the leading DNA strand (Figure 1) [2,15]. This process is also referred to as DNA editing by APOBECs (Figure 1). Several APOBEC proteins have been shown to also edit C-to-U in RNA molecules in vitro [16,17,18]. The best characterised members of the APOBEC family are APOBEC1, an editor of apolipoprotein B mRNA, and activation-induced deaminase (AID), a DNA editing enzyme that is a key regulator in somatic hypermutation and class-switch recombination that enables immunoglobulin diversification in vertebrates [19]. APOBEC proteins have been discovered in all vertebrates, where they provide innate immunity against endogenous and exogenous retroelements [13,20,21,22]. 

**Figure 2:**
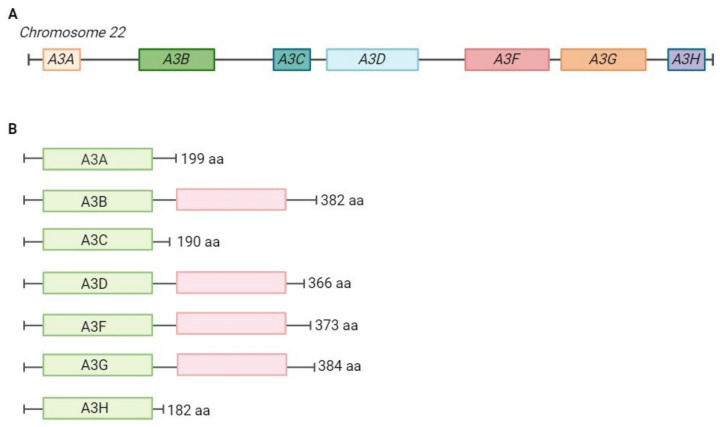
Genomic organisation of the APOBEC3 locus and domain organisation of APOBEC3 proteins. (**A**) APOBEC3 locus on chromosome 22. APOBEC3 genes evolved through complex gene duplication and positive selection [2,23]. (**B**) Domain organisation of the seven human APOBEC3 proteins. APOBEC3 proteins have either one or two zinc-binding domains. In four APOBEC3s (A3F, A3B, A3D, A3F, A3G) the first domain (in green) is catalytically active, the second domain (in red) is catalytically inactive. The length of the protein is shown in the number of amino acids. aa—amino acids.

The evolutionary origin of APOBECs predates the emergence of vertebrates. The enzymes probably evolved from the tRNA adenosine deaminase (TAd)/adenosine deaminase, tRNA–specific 2 (ADAT2) deaminases [20]. APOBEC3 proteins are specific to mammals, where their copy number highly varies. Primates have seven APOBEC3 genes (A3A, A3B, A3C, A3D, A3F, A3G, A3H), while mice have only one (mA3) (Figure 2) [20,23]. All seven human members of the APOBEC3 family differ in tissue-specific expression, cellular localisation, substrate specificity, editing context specificity, and other characteristics [2,15]. The active site of APOBEC3 proteins is characterised by a conserved zinc-binding motif (Cys/His)-Xaa-Glu-Xaa_23–28_Pro-Cys-Xaa_2–4_-Cys [13,24]. Four human APOBEC3 proteins (A3B, A3F, A3DE, A3G) and the mouse APOBEC3 (mA3) have two cytidine deaminase domains, whereas A3A and A3C have only one (Figure 2). A second domain has probably evolved to separate the nucleic acid binding function from the domain of enzymatic activity. 

APOBEC3s were initially discovered as antiviral factors in HIV-1 infection [9], but were later identified as general antiviral factors that block replication of various RNA viruses, DNA viruses and retrotransposons [1,2,13,25,26,27]. Generally, APOBEC proteins can inhibit the replication of viruses by deaminating cytosine to uracil in the viral DNA during replication. This action results in hypermutation of the viral genome, leading to a loss of viral fitness and, in many cases, to the inactivation of the virus. AID/APOBECs have also been shown to be involved in epigenetic regulatory mechanisms, with active demethylation of 5-methylcytosine (5mC) being mediated by AID deamination of 5mC [28,29].

The best-studied antiviral effect of APOBEC3 is the inhibition of HIV-1 replication [2,9]. The first member of the APOBEC3 family to be discovered as a host restriction factor against HIV-1 viral infectivity factor (Vif)-deficient virus was human A3G [24]. A3G is incorporated into the virions, where it causes extensive deamination of C-to-U in the newly synthesized viral cDNA during viral replication [24]. Other early steps of viral DNA synthesis and/or integration may also be affected [30]. Several groups have shown that both A3F and A3G restrict viral replication of HIV-1 by being incorporated into virions via interactions with the viral nucleocapsid (NC) and viral RNA [1,31]. After viral infection of the target cells and uncoating, APOBEC3s localise the viral RNA and cause excessive C-to-U mutations during reverse transcription [32,33]. 

After extensive research into the inhibition of HIV replication, studies quickly expanded to the majority of viruses. Different members of the APOBEC3 family have been shown to inhibit a variety of viruses—retroviruses—as follows: HIV-1ΔVif (ssRNA-RT) [9], murine leukaemia virus MLV (ssRNA-RT) [34], mouse mammary tumour virus (MMTV) (ssRNA-RT) [25], foamy virus (ssRNA-RT) [35,36], and single-stranded RNA hepatitis C virus (ssRNA) [37]. Among the DNA viruses, inhibition of replication has been demonstrated for single-stranded DNA viruses, including parvoviruses (ssDNA) [38] and adeno-associated virus (ssDNA) [39] and for dsDNA viruses, including the hepadnavirus hepatitis B (dsDNA-RT) [40] and the human papillomavirus (dsDNA) [41]. Traces of C-to-U editing in the ssRNA coronavirus SARS-CoV-2 suggest that APOBEC3s could also cause mutations in SARS-CoV-2 [17,42]. Recently, APOBEC3 mutations were discovered in the zoonotic dsDNA virus mpox (monkeypox virus), which helped to explain an unusually rapid evolution of the poxvirus. The transmission of the mpox virus from human to human has accelerated its evolution [43]. The importance of APOBEC3 proteins in viral restriction has also been demonstrated in vivo. We have shown that mice lacking the APOBEC3 gene are more susceptible to infection with mouse mammary tumour virus (MMTV) than wild-type mice [25]. Sequence analyses of HIV-1 proviral DNA isolated from infected patients revealed a high level of G-to-A mutations, suggesting that they may arise from APOBEC3 cytidine deamination [44]. In addition, evolutionary analyses of APOBEC3 proteins indicate that they have been under strong selective pressure over the last 30 million years. Additionally, polymorphisms have been found that contribute significantly to their antiviral efficacy [45,46,47].

APOBEC-induced mutations have been be computationally explored in viral, retroelement or genomic sequences [15,45]. Different APOBECs deaminate C to U in different nucleotide contexts. For example, A3G favours the CC sequence, A3F TTC (underlined deaminated nucleotide) [15]. In addition to the nucleotide context, the secondary structure of DNA is also crucial for the efficiency of DNA editing. The secondary structure of DNA controls the editing of “hotspots” in genomic DNA [48]. Interestingly, the APOBEC mutation pattern (G-to-A mutations) has been detected in numerous DNA and RNA viruses and retrotransposons [17]. We have identified G-to-A edits in non-mammalian vertebrate retroelements, suggesting that APOBEC editing predates mammals [49]. Traces of APOBEC mutations have been identified in the genomes of vertebrates, implying that APOBECs were important players in the restriction of retroelements and influenced their evolution [15,45,49]. Similar to the editing-independent inhibition of viral replication [50], the restriction of retrotransposition could also be editing-independent [14,46,49,51,52]. 

A comprehensive study of 33,400 viral genomes revealed that at least 22% of currently known viruses are affected by APOBEC3 selection pressure [8]. Interestingly, the DNA viruses Papillomaviridae and Polyomaviridae showed the highest APOBEC3 footprint. The authors hypothesise that small DNA viruses require APOBEC3 editing to drive their evolution. Analysis of the genomes of 36 RNA viruses revealed that 15–20% of the sequence variability in RNA viruses is due to C-to-U editing, indicating the importance of APOBEC editing in the evolution of RNA viruses [17]. The overrepresentation of C-to-U changes varies between RNA viruses and reaches 7.5-fold in SARS-CoV-2. The interaction between APOBEC3 proteins and viruses has a significant impact on viral evolution. The mutagenic activity of APOBEC3 can drive viral diversification and adaptation, potentially leading to the emergence of drug-resistant or immune-volatile variants. However, the balance between beneficial and harmful mutations is delicate; excessive hypermutation can lead to virus attenuation or eradication. Therefore, the activity of APOBEC3 proteins can exert selection pressure on virus populations and influence their evolutionary trajectory.

## 3. Restriction of DNA Viruses by APOBEC Proteins 

### 3.1. Hepatitis B Virus

Hepatitis B virus (HBV), a hepadnavirus, is a well-studied example of APOBEC3-mediated restriction of DNA viruses [53,54]. Chronic HBV infection is associated with the development of liver cancer and is considered the most important cause of the high incidence of liver cancer in the Asian population [55]. HBV promotes the development of liver cancer mainly through indirect inflammatory damage and through the direct carcinogenic effect of the viral oncoprotein BHx [56]. HBV has a double-stranded DNA, which is replicated through RNA intermediates. After uncoating in the cytoplasm of liver host cells, the HBV genome is transported into the nucleus. The genomic DNA (relaxed circular, partially double-stranded DNA, or rcDNA) is first converted to a molecular template DNA (covalently closed circular DNA, or cccDNA). Similar to retroviral replication, the HBV DNA is transcribed into a pregenomic RNA (pgRNA) and viral protein mRNAs. During assembly, the viral RNA is incorporated together with other factors, including members of the APOBEC3 family, such as A3G. After RNA incorporation, the viral genome is synthesized in several steps, leading first to the synthesis of the (−) and then the (+) DNA strand (the HBV cell cycle is reviewed in [57]). The reverse transcriptase is covalently bound to the 5′ of the (−) strand and cannot fully transcribe the (+) strand, leaving a short sequence of single-stranded DNA as a possible target for the APOBEC3 proteins [54]. Indeed, APOBEC3-induced HBV genome mutations have been shown to occur primarily when the HBV RNA in the viral capsid is reversibly transcribed into (−) DNA [58]. 

In vitro studies and analyses of clinical samples show that APOBEC3 proteins can substantially limit HBV infection, either by hypermutation of viral DNA or by editing-independent mechanisms. Five of the seven APOBEC3 genes were upregulated in cirrhotic liver samples, with G-to-A mutations observed to be consistent with the substrate preference of A3G [59]. A similar expression pattern has been observed in another study of four patients with high viremia [60]. A weak prevalence and preferential (+) strand distribution of A3G-induced mutations on the HBV DNA has been found in HBV-infected individuals with or without hepatocellular carcinoma (HCC) [61]. Although A3G levels are usually low in primary hepatocytes, they can be substantially upregulated during chronic infection in response to interferon-α [62]. 

Several cell culture models provided a much broader wealth of data that indicate a role for several APOBEC3 family proteins in viral restriction. A3B, A3G and A3F appear to be the major hypermutators of HBV DNA in vitro, causing both G-to-A and C-to-T substitutions [60,62]. A3A and A3C are also thought to be involved [60,63,64], although the in vitro evidence for their deamination activity in HBV DNA appears less compelling. A3G and A3C [65], but also A3B and A3F [62], can bind to the HBV core protein and thus possibly package into the viral cores in the cytoplasm. Interestingly, in an endogenous setting of liver cells treated with interferon-α or an antibody to cross-link the lymphotoxin β receptor, upregulation of A3A and A3B was observed, leading to G-to-A substitutions and a reduction in viral cccDNA replication intermediates [66]. It appears that A3A/A3B-induced mutations affecting the early stages of the lytic life cycle, specifically the immediate early and early genes, are more likely to be lethal to the virus than mutations in late genes [67]. 

Although almost all APOBEC3 members can deaminate HBV DNA in vitro, this is not always reflected in viral restriction as measured by absolute DNA levels, hepatitis B surface antigen (HBsAg) levels or HBV decline [68]. A good example is A3D, which shows a proviral phenotype by forming a complex with A3F and A3G, thus reducing their packaging in the HBV virions [69]. A3F, in turn, can reduce HBV DNA levels; however, its infrequent DNA editing and inability to edit viral RNA suggest that it plays only a minor role in viral restriction [70], with no or very little G-to-A hypermutation. A similarly negligible anti-HBV effect has also been reported for A3A, despite its ability to induce HBV genome hypermutation [71]. The A3H/hapII splice variant [72] and A3B both show anti-HBV activity, the latter possibly inhibiting the progression of chronic hepatitis B [73]. Of all APOBEC3 proteins, A3G appears to have the most potent restrictive effect. A3G has been shown to block replicative HBV intermediates in liver cell lines in a dose-dependent manner [74] and to increase the risk of advanced liver disease in HBV carriers lacking the A3G H186R variant [75]. Although G-to-A hypermutation in the context characteristic of A3G is detected at low levels in clinical samples, A3G appears to limit HBV infection by suppressing viral pregenomic RNA packaging rather than inducing G-to-A hypermutation [40]. A3C is generally considered less important for HBV viral restriction. However, a recent report has shown increased restriction activity and hypermutation frequency of the natural APOBEC3C variant S188I in an HBV-replicating cell culture model [72].

Deamination activity is the most apparent restriction activity of APOBEC3 proteins, but it is not the only one. Inhibition of HBV gene transcription and viral particle generation also has a significant impact on HBV infection [76]. A3B reduced HBV replication in hepatocytes by inhibiting heterogeneous nuclear ribonucleoprotein K (hnRNP K)-mediated transcription and expression of HBV genes as well as HBV core-associated DNA synthesis [76]. In contrast, A3G does not interfere with the early steps of HBV viral morphogenesis but impedes the production of infectious HBV viral particles. Total RNA and DNA production has been shown to not become altered in an A3G-dependent manner [62], though a reduction of full-length HBV DNA has been observed in the presence of A3G [70]. A3G also inhibits HBV reverse transcriptase within core particles [70,77], and reduces the replicative capacity of cccDNA, including reduction in nucleocapsid DNA, precore mRNA, and secreted viral particle-associated DNA in extended culture [78,79].

### 3.2. Human Papillomaviruses

Double-stranded DNA viruses are also a viable target for APOBEC3, particularly during transcription or viral genome replication involving transient single-stranded DNA. Characteristic G-to-A mutations found in the HPV16 genome in the precancerous cervical biopsies were the first indication that APOBEC3s may be involved in HPV infection [41]. Human papillomaviruses (HPV) are small, non-enveloped DNA viruses that infect skin and mucosal epithelial cells and cause hyperproliferative lesions. The transfer of the viral genome into the host cell nucleus requires complex intracellular trafficking and recruitment of the various components of the host cell transport machinery [80,81,82]. As the infected cells differentiate, the viral genes are expressed in a coordinated manner, eventually leading to the release of new virion particles in the terminally differentiated epithelial cells [82]. If not cleared by the immune system, persistent infection with high-risk HPV genotypes often leads to the integration of the HPV genome into the host chromosomes. At the same time, the HPV oncoproteins E6 and E7 contribute to the progression of cell transformation and cancer development [83]. Upregulation of A3A and A3B has been observed in clinical samples of cervical cancer and HPV-positive head and neck squamous cell carcinoma (HNSCC) [84,85,86]. Subsequent in vitro studies in keratinocytes have shown that both HPV oncogenes, E6 and E7, regulate the expression of A3A and A3B by either transcriptional or post-transcriptional mechanisms [87]. HPV E6 enhances the upregulation of A3B protein in keratinocytes by activating a specific region in the A3B promotor, induced by the zinc finger protein ZNF384 and the TEA domain transcription factor 4 (TEAD4) [88,89]. A3B can also be upregulated by eliminating the inhibitory activity of p53 and pRb exerted by high-risk HPV oncoproteins. HPV E6-dependent degradation of p53 prevents induction of p21 (CDKN1A) and recruitment of the repressive DREAM complex to the A3B gene promoter [90]. On the other hand, HPV E7 efficiently degrades pRB and thus augments the E2F functions of transcriptional activation, leading to the upregulation of A3B mRNA [84]. A post-transcriptional mechanism is involved in the upregulation of A3A protein by high-risk E7. HPV E7 stabilises A3A protein levels by interfering with cullin 2-dependent protein polyubiquitination and degradation [91].

Hyper-edited HPV1a and HPV16 genomes have been discovered in plantar warts and HPV16 precancerous cervical biopsies, and were correlated with the expression levels of A3A, A3B and A3H [41]. In vitro studies with overexpressed APOBEC3 proteins and HPV plasmid DNA have confirmed that APOBEC3 is involved in editing the HPV genome [41,92]. In tissue samples from cervical dysplasia, mutations characteristic of APOBEC3 were most frequently detected in the long control region (LCR) [93], which contains a viral origin of replication and the early promoter. Although this has not been confirmed experimentally, mutations in LCR could affect HPV replication and transcription of viral genes. Exogenous expression of APOBEC3 proteins showed notable editing of the HPV16 E2 gene by A3G and partially A3A, while HPV16 episome levels remained unaffected [92]. The APOBEC3 mutations were most heavily clustered in the C-terminal DNA-binding domain of E2 involved in transcription and replication of the viral genome [94]. The second possibility is that the uracil bases in the E2 gene generated by APOBEC3s may enhance the DNA strand breaks, leading to viral DNA integration. Hypermutation in the HPV16 E2 gene has also been observed in cervical intraepithelial neoplasia, confirming its importance during natural infection [95]. On the other hand, the HPV oncoprotein E7 is highly conserved in cancer samples, a phenomenon that is associated with higher carcinogenicity. However, many non-silent variants observed in E7 are consistent with APOBEC3-induced mutations in HPV-positive precancerous and cancerous tissues, potentially reducing HPV16 E7 carcinogenicity [96]. A higher mutational load in HPV16-infected cervical tissue has indeed been found to be associated with enhanced viral clearance, suggesting that APOBEC3-induced mutations may reduce persistence and disease progression in the HPV-infected hosts [97]. 

The mechanism of the A3A restriction of HPV replication has been deciphered by Warren et al. [84]. They demonstrated that the infectivity of HPV virions produced in the presence of A3A is substantially reduced compared with A3B or A3C proteins in human keratinocytes and that the restriction of viral infection was editing dependent. No evidence of A3-mediated DNA editing was found in known APOBEC3-editing hotspots within virion-associated DNA, warranting further studies to elucidate the mechanism of APOBEC3-associated HPV viral restriction.

### 3.3. Human Polyomaviruses

Human polyomaviruses (HPyV) are a group of non-enveloped viruses with a small double-stranded circular DNA. After entering the host cell, HPyV travels to the cell nucleus where the virus utilises the host cellular replication machinery to replicate its DNA. The viral DNA consists of a non-coding control region (NCCR) and an early and a late transcription region. The early region contains coding sequences for large and small T antigens, which play a role in viral replication and have been associated with viral chromosomal integration. The late region generally encodes three viral structural capsid proteins, VP1, VP2 and VP3 [98]. 

BK polyomavirus (BKPyV) and JC polyomavirus (JCPyV) are the causative agents of transplant-related kidney diseases. BKPyV has also been linked to the aetiology of bladder and kidney cancers, although the mechanism is not yet entirely clear [99]. A recent report has shown that the expression of the large T antigen of BKPyV upregulates A3B in cultured cells. On the other hand, the same study discovered an underrepresentation of A3B target sites in BKPyV DNA [100]. It appears that the truncated T antigens of BKPyV and JCPyV trigger overexpression of A3B through its retinoblastoma (RB)-interacting motif, which modulates the binding of E2F family transcription factors to promote A3B expression [101]. APOBEC3 expression has also been detected in immunohistochemical analyses of renal biopsies from kidney transplant recipients with BKPyV-related nephropathy and subsequent renal carcinoma. Despite the cross-reactivity of the anti-A3B antibodies, nuclear staining strongly suggests A3B as the most likely upregulated enzyme [102]. BKPyV variants from the same samples have indeed shown various mutations in the major capsid protein VP1, which is consistent with the A3B signature. Mutations in the VP1 surface loops conferred relative resistance to serum antibody-mediated neutralization and engaged a different spectrum of cell surface glycans for cell entry [102]. Three mutational signatures in the BKPyV VP1 gene were consistently observed in urine, serum, and kidney biopsy samples from kidney transplant recipients with persistent viral replication, two of which were consistent with A3A/B activity [103]. 

Merkel cell polyomavirus (MCPyV) is associated with the development of highly lethal Merkel cell carcinomas (MCC) [104]. Mutations of MCPyV T antigens are important pathologic events in virus-positive Merkel cell carcinomas, many of which result in large T (LT) truncation [105]. In MCPyV-positive MCC, a strong APOBEC3 mutational signature has been observed in the MCPyV LT gene, making APOBEC3 deamination events a plausible cause of LT truncation mutations. A much lower but statistically significant somatic hypermutation activity has also been detected in the regulatory region of MCPyV [106]. Sequence analysis of MCPyV has revealed an underrepresentation of the characteristic A3A and A3B motifs in the 3′-region of the large T antigen-coding sequence [107]. Furthermore, A3A, A3B, and A3G were able to introduce APOBEC3-specific mutations into episomal MCPyV genomes in MCPyV-replicating 293-derivative cells [107]. In a recent study in which the genomes of 33,400 human viruses were analysed for depletion of APOBEC3-favoured motifs, BKPyV, JCPyV, and MCPyV were all found to have a genome-wide APOBEC3 footprint present on both DNA strands [8]. The magnitude of the APOBEC3 footprint appears to be lower in MCPyV.

### 3.4. Gamma Herpesviruses

Herpes viruses are another group of double-stranded DNA tumour viruses. Epstein–Barr virus (EBV) and Kaposi’s sarcoma-associated herpesvirus (KSHV) are large-enveloped gamma herpesviruses known for their ability to establish latency in host cells. EBV infects epithelial and B cells, establishing latency primarily in B cells [108]. B cells are interesting because they have an upregulated activation-induced cytidine deaminase (AID), which drives the somatic hypermutation of immunoglobulins and class switch recombination in the germinal centres as part of the adaptive immune responses [109]. Viruses that infect B cells are therefore susceptible to restriction by AID. Gene expression data from the GTEx consortium indeed show increased mRNA levels of AID and all APOBEC3 enzymes except A3A in EBV-transformed lymphocytes [110]. EBV is associated with several malignancies, including Burkitt’s lymphoma, nasopharyngeal carcinoma, Hodgkin’s lymphoma, gastric carcinoma, and breast cancer [111]. Several recent studies have shown that AID/APOBEC3 enzymes may contribute to the development of these malignancies [110]. Kaposi’s sarcoma-associated herpesvirus (KSHV), on the other hand, can infect several cell types, including B cells, epithelial cells, endothelial cells, monocytes, and keratinocytes, and can establish latency in B cells and endothelial cells. KSHV has been associated with the development of Kaposi’s sarcoma, multicentric Castleman’s disease and primary effusion lymphoma in immunocompromised patients [110].

After entering the host cell, the herpesvirus DNA is transported into the cell nucleus, where it forms a stable extrachromosomal episome. This process also determines whether the initial infection proceeds to a lytic or latent replication mode. In the latency, a limited number of viral genes are expressed, and the viral DNA is segregated into new cells during the cell decision. The lytic phase is characterized by the coordinated production of numerous viral proteins, which enable viral DNA replication, assembly of viral particles in the nucleus and cytoplasm, and subsequent release from the host cell [111]. Herpesvirus genomes are considered exceptionally stable and have a relatively low frequency of the G-to-A and C-to-T mutations that are characteristic of APOBEC editing. This has been attributed to the chromatinisation of viral DNA in the nucleus, which disguises the viral genome as cellular DNA [112]. The second reason could be the high-fidelity DNA replication mechanisms that are utilized by herpes viruses [113]. Many studies have suggested that human gamma herpesviruses are under evolutionary pressure and are subject to AID/APOBEC3 hypermutation. Reports on the APOBEC3 mutational signature in herpesviruses showed a strong underrepresentation of the 5′-TC motif, a substrate motif for A3A and A3B, in both EBV and KSHV. This contrasts with the overrepresentation of the CCC motif characteristic of A3G [110]. In EBV, a depletion of 5′-TC and an enrichment of 5′-TT were discovered in specific genomic regions, including the viral origin of replication (ori) [7,110]. In contrast with papillomaviruses and polyomaviruses, which have genome-wide APOBEC footprints, the APOBEC footprints in gamma herpesviruses are restricted to concise segments, mostly belonging to lytic origins of replication [8]. The nature of APOBEC hotspots in the EBV genomes and the intracellular distribution of APOBEC3 proteins suggest that A3B may be the primary deaminating APOBEC3 enzyme in EBV due to its predominant nuclear localization and high relative expression in EBV-tropic tissues [110]. AID may also be involved in EBV mutagenesis by deamination of the characteristic 5′-WRC motifs in the viral genomes within B cells [8,110].

EBV and KSHV have evolved to limit the hypermutation of the AID and APOBEC3 enzymes. The EBV latency protein EBNA-2 can inhibit AID expression [114], while AID translation is inhibited by the expression of endogenous miR-155 and miR-93 [115]. KSHV uses the protein LANA-1 to recruit uracil DNA glycosylase 2 and neutralize the mutagenic effect of AID. On the other hand, AID translation is inhibited by the expression of endogenous miRNAs K12–11 and K12–5 [116]. Gamma herpesviruses also employ a unique APOBEC3 counteracting mechanism by binding, inhibiting and relocalising nuclear APOBEC3 enzymes to the cytoplasm through the viral ribonucleotide reductase (RNR) [117,118]. One example is the BORF2 protein of EBV, which inhibits A3B deaminase activity and re-locates it far away from the viral replication centres [7]. The potential viral restriction mechanisms of AID/APOBEC3 have hardly been investigated in in vitro studies and animal models of herpesvirus infection. It has been shown that overexpression of A3C, though not A3A, A3G or AID, can lower herpes simplex virus 1 (HSV-1) titers in cell culture models [119]. Another study has confirmed that overexpression of A3A and A3B has almost no effect on the infectivity of HSV-1 virions [38,117]. Both observations can be connected to the redundancy of APOBEC3 counteracting mechanisms in alpha herpesviruses. Further studies are needed to characterise the exact mechanism of viral restriction by AID/APOBEC3 proteins and to determine whether similar mechanisms exist in gamma herpesviruses such as EBV and KSHV.

The summary of how different APOBEC3 members contribute to DNA virus restriction, viral genome editing, and host genome editing is presented in Table 1.

## 4. How APOBEC3 Proteins Contribute to the Oncogenicity of DNA Viruses?

In humans, an APOBEC3 mutation pattern (G-to-A mutations) has been detected in various cancers, and DNA hyper-editing has been associated with upregulation of the A3B protein [121,122,123]. Indeed, broad genomic studies have revealed the APOBEC3 mutations as the second most frequently detected mutational signature in human cancer tissue, next to ageing-related signatures [27,120,123,124,125,126]. While earlier studies have suggested A3B as the primary source of APOBEC3 mutational signatures in cancer [27,121,123], recent studies have shown that A3A-like mutations are ten times more frequent than A3B-like mutations [121,127,128]. A3A preferentially mutates the nucleotide sequence YTCN, whereas A3B deaminates the nucleotide sequence RTCN [29]. Deletion of A3A in human cell lines has been shown to lead to a decrease in the APOBEC3 mutational signature, demonstrating the importance of this mutational agent for cancer cell genome evolution [125]. The important role of A3A and A3B in triggering C-to-T and C-to-G mutations in cancer has also been demonstrated via experiments with a genetically modified HAP1 cell line [129]. The improvement of bioinformatics tools has led to the discovery of clustered and non-clustered APOBEC3 mutations, further explaining the role of APOBEC3 mutations in cancer development [125]. Recently, the importance of DNA hairpin structure for A3A substrates has been uncovered, explaining that A3A can also deaminate the less favourable VpC sequence (compared with TpC) and cause canonical mutations in trans, leading to hotspots and cancer development [127]. A comprehensive study of 2583 whole genomes from 30 cancer types showed APOBEC3 kataegis in extrachromosomal DNA, highlighting the impact of APOBEC3 mutagenesis beyond the traditional boundaries of chromosomes and opening new implications for the role of APOBEC3 in tumorigenesis [125]. 

Cancers with a strong APOBEC3 signature include HPV-associated cancers, namely cervical cancer and HPV-positive HNSCC [122,128]. A whole-genome and exome sequencing analysis conducted by the Cancer Genome Atlas revealed that APOBEC3 mutagenesis is the predominant source of somatic mutations in cervical cancer [130]. Similar data had been previously obtained for HNSCC, where significantly higher overall levels of APOBEC3 signature mutations were found in HPV-positive cancers [128]. Cervical cancer has two primary mutational signatures. The first is the APOBEC signature, while the second belongs to the age-related C-to-T mutations at methylated cytosine–guanine (CpG), otherwise known as signature 1b and which is a common pattern in many cancers [4]. APOBEC-characteristic mutations are significantly enriched in cancer tumours with high expression of HPV oncoproteins E6 and E7 (HPV-active tumours), whereas signature 1b is characteristic of HPV-inactive tumours [131]. As in some other cancers, A3A-like mutations are the most frequent mutations in cervical cancer and HNSCC [126]. A3A mRNA expression was found to be increased by the high-risk HPV oncoprotein E7, while A3B mRNA expression is upregulated by both HPV oncoproteins E6 and E7 [84,132]. The increased A3B expression is associated with abrogated p53-mediated suppression of A3B expression by the viral oncoprotein E6/E7 [90]. 

**Figure 3:**
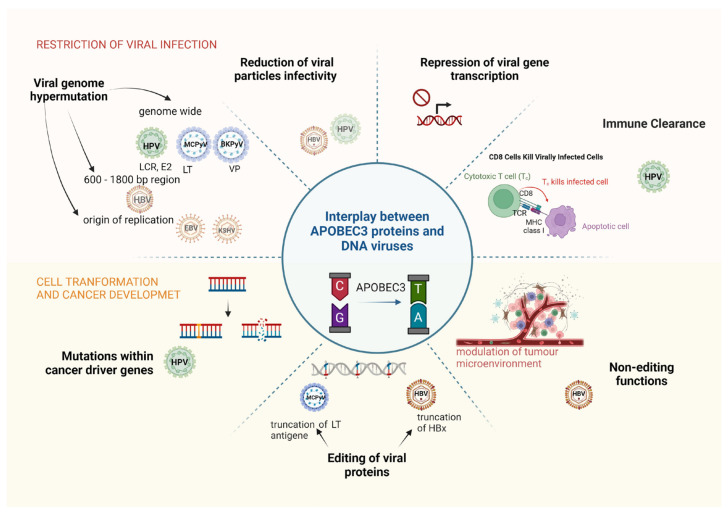
Dual role of APOBEC3 proteins in viral restriction and oncogenicity of DNA tumour viruses. Based on the available studies, different roles of APOBEC3 are attributed to different DNA tumour viruses. Please note that particularly affected gene regions are shown in otherwise genome-wide mutations of HPV, MCPyV and BKPyV. Abbreviations: HPV, human papillomaviruses; HBV, hepatitis B virus; EBV, Epstein–Barr virus; MCPyV, Merkel cell polyomavirus; BKPyV, BK polyomavirus; LCR, long control region; LT, large T antigen; VP, major capsid protein VP; HBx, hepatitis B virus X protein.

APOBEC-mediated somatic mutations contribute to the development of HPV-associated cancers by generating driver mutations in numerous cancer “hotspot” genes (Figure 3). HPV-associated cancers exhibit a recurrent somatic mutation at two helical domain hotspots in the oncogenic driver gene *PIK3CA*, which is involved in the regulation of cell growth, proliferation, differentiation, glucose metabolism, protein synthesis and apoptosis. *PIK3CA* is frequently mutated in cervical cancer [133] and in HPV-positive HNSCC [134] and is associated with a poorer prognosis than cancers with wild-type *PIK3CA* [135]. Whole genome sequencing of paired samples of cervical carcinoma and normal tissue exposed APOBEC3-associated mutations in several other genes, such as *FBXW7*, *EP300*, *MAPK1*, *TP53*, *ERBB2*, and *NFE2L2* [133]. Interestingly, there are many similarities in the mutations found in HPV-driven cancers, suggesting that the mechanisms are likely similar in other HPV-associated cancers for which it is difficult to conduct large genomic studies. For example, a recent study of HPV-positive squamous cell carcinomas at four anatomical sites found no statistically significant differences in gene mutations of 48 candidate genes, including *PIK3CA*, *EGFR*, *NOTCH1*, and *KRAS* [136]. This is very promising, as targeted treatment strategies can be widely applied. 

APOBEC3-associated mutagenesis might not only promote carcinogenesis but could also trigger the formation of neoantigens and thus the activation of the host immune system. APOBEC3 mutations have been associated with immune activation and host immune cell infiltration in some cancers, including HNSCC [137]. However, high intra-tumoral heterogeneity due to a high mutational burden may also lead to T cell exhaustion and poor survival [138], which is supported by the observation that a T cell exhaustion marker, PD-1, is upregulated in HPV-positive HNSCCs [139]. Nonetheless, HPV-positive HNSCCs are characterised by higher A3A, A3B and A3H expression, higher mutational burden, a positive correlation between A3H and CD8+ T-cell infiltration, and better overall survival associated with higher A3G or A3H levels [87]. How APOBEC3 mutagenesis affects the immune response and disease progression in other HPV-associated cancers is far less clear and may be more specific to cancer types. 

For cancers associated with other DNA viruses, the evidence of APOBEC3-related genomic mutations in the host is far less consistent. A recent study of 240 gastric cancer samples showed a positive correlation between APOBEC3 expression and APOBEC3-related mutational burden in EBV-positive gastric cancer [140]. A significantly higher proportion of APOBEC3 signature mutations in *GFR3/PIK3CA* has also been detected in polyomavirus (PyV)-positive bladder tumours [120]. In contrast, an APOBEC3 mutational signature is absent in MCPyV-positive MCC [141,142], which is very interesting given the APOBEC3 signature mutations in PyV-positive bladder tumours. A similar negligible APOBEC3-associated mutational pattern has been found in hepatocellular carcinoma (HCC), which is strongly associated with HBV infection [4,143].

Both cancers exhibit upregulation of APOBEC3 proteins, but this could also contribute to cancer development by mutation of the viral genome or by non-editing functions of the APOBEC3 proteins. A recent report has shown that APOBEC3 cytidine deaminases are a plausible cause of viral large T truncating mutations in MCPyV-positive MCC, an important pathologic event in aggressive carcinomas with poor prognosis [106]. A large number of APOBEC3 proteins has been detected in HBV-associated HCC. However, the role of APOBEC3 in tumorigenesis and disease progression in HCC is not yet well understood. Expression analysis has revealed overexpression of A3B, A3D, A3F and A3H in HCC tumours compared with non-tumour tissue [144]. The correlation of A3 expression with clinicopathologic features and disease progression suggests that A3G and A3F, but probably also A3B, are risk factors for HCC development and survival [144,145]. The upregulation of A3B may contribute to viral cccDNA editing in HCC patients, but may also generate the HBx truncation mutants that increase the proliferative ability of neoplastic cells [53,146]. In addition to its deamination activity, A3B may act as an immunomodulatory molecule that facilitates a favourable immune microenvironment during cancer progression [147]. A positive feedback loop has been reported for A3B and interleukin-6 (IL-6) in HCC cells, suggesting that A3B may thus support the recruitment of myeloid-derived suppressor cells (MDSCs) and tumour-associated macrophages (TAMs) into the tumour microenvironment [148]. In another study, upregulation of A3B by non-classical nuclear factor-κB (NF-κB) signalling promoted HCC growth in immunocompetent mice. This was associated with A3B-dependent upregulation of the chemokine CCL2 and the increase in the immunosuppressive MDSCs and TAMs [149].

## 5. Conclusions and Perspectives

The APOBEC/AID family of cytidine deaminases became fascinating enzymes when their role in the restriction of retroviruses, retrotransposons and DNA viruses began to be investigated twenty years ago. Later, another role of APOBEC3 in somatic hypermutation of host genomes became known, a role that is surprisingly widespread in various types of cancer. APOBEC3 proteins play a dual role; while DNA editing activity is an essential component of the innate immune response to viral infections and restricts retrotransposons, it may also contribute to the pathogenesis of persistent viral infections and cancer progression. Expression of APOBEC3 proteins due to viral infection is likely not the only reason for APOBEC3’s off-target activity, as induction of the APOBEC pathway and its contribution to carcinogenesis is found in many cancers not associated with infectious agents, including breast cancer and serous ovarian cancer. However, in viral infections, increased expression of APOBEC3 enzymes most likely promotes APOBEC3-associated somatic mutagenesis. It is currently not clear when APOBEC3 mutations begin to accumulate in the host genome during persistent infection and whether APOBEC mutations occur before or after non-APOBEC mutations. The second unresolved question is what significance the APOBEC3 signature mutations have in different types of cancer and whether they are driver mutations or mere passenger mutations in cancer progression. The latter has been increasingly mentioned in recent years, although some recent studies support the idea that DNA editing plays a crucial role in somatic mutagenesis and cancer progression when host cancer driver genes or viral oncogenes are involved.

There are also many unanswered questions about the role of APOBEC3 in the infection and carcinogenicity of DNA viruses. After initial enthusiasm when APOBEC3 was associated with most of human DNA viruses, progress seems to have slowed down in recent years. Although APOBEC3 proteins are frequently upregulated in the infected tissues and APOBEC3 mutations are detected in viral genomes, we do not yet know the exact antiviral mechanisms of APOBEC3 for most DNA viruses. This may be partly related to active viral actions against APOBEC3 proteins, as in the case of gamma herpesviruses, or the mechanisms may be much more subtle than we thought. Another possibility is that the restriction is indirectly related to the immunological milieu in which the infection develops. A similar observation has been made in the progression of HCC, where upregulation of A3B leads to the recruitment of immunoinhibitory cells. Studies on the role of APOBEC3 in the restriction of DNA tumour viruses are still urgently needed, considering their causal link to human cancer. The use of APOBEC3 as therapeutic targets may not be very close, but understanding their antiviral mechanisms could help to reduce the persistence of viral infection as an important risk factor for cancer development and reduce the off-target effects of APOBEC3s on the host genome during their prolonged expression.

## Figures and Tables

**Table 1:** The role of the different APOBEC3 proteins in viral and host genome editing.

DNA Tumour Virus		Viral Genome Editing by A3/AID ^a^	Restriction of Infection	A3 Expression in Tumours	A3 Mutational Signatures in Cancer	Mutations in Cancer Driver Genes/Viral Oncogenes	References
Hepadnaviruses	HBV	Localised: A3A, A3B, A3C, A3D, A3F, A3G, A3H	A3B, A3C, A3F, A3G, A3H/hapII	A3B, A3D, A3F, A3G, A3H	Low	Unknown/HBx	[6,59,62,63,64,70,72,73,74,76,77,78,119,120]
Papillomaviruses	HPV	Genome-wide: A3A, A3B, A3H	A3A	A3A, A3B	Strong: A3A, A3B	PIK3CA, FBXW7, EP300, MAPK1, TP53, ERBB2, NFE2L2, KRAS,…	[41,84,85,86,92,93,95,121,122,123,124]
Polyomaviruses	MCPyV	Genome-wide: A3A, A3B, A3G	Unknown	A3A, A3B and A3H	Low	Unknown/LT antigen	[99,106,107,125]
	BKPyV	Genome-wide: A3A, A3B	Unknown	Unknown	Unknown	GFR3/PIK3CA ^b^	[100,102,103,126]
Gamma-herpesviruses	EBV	Localised: A3B (A3H), AID	Unknown (A3C in HSV-1)	A3B, A3C, A3D, A3F, A3G, A3H	EBV-positive breast and gastric cancers	PIK3CA and some other oncogenes	[7,8,110,119]

A3 members and driver genes with highlighted significance are marked in bold. ^a^ Specific A3 proteins are predicted in some cases based on their preferred motifs. ^b^ Data from PyV-positive bladder cancer. Abbreviations: A3, APOBEC3; HPV, human papillomavirus; HBV, hepatitis B virus; EBV, Epstein-Barr virus; MCPyV, Merkel cell polyomavirus; BKPyV, BK polyomavirus; HSV-1; herpes simplex virus 1; HBx, hepatitis B virus X protein; LT, large T.

## Data Availability

Not applicable.
